# Molecular Predictors of 3D Morphogenesis by Breast Cancer Cell Lines in 3D Culture

**DOI:** 10.1371/journal.pcbi.1000684

**Published:** 2010-02-26

**Authors:** Ju Han, Hang Chang, Orsi Giricz, Genee Y. Lee, Frederick L. Baehner, Joe W. Gray, Mina J. Bissell, Paraic A. Kenny, Bahram Parvin

**Affiliations:** 1 Life Sciences Division, Lawrence Berkeley National Laboratory, Berkeley, California, United States of America; 2 Department of Developmental and Molecular Biology, Albert Einstein College of Medicine, Bronx, New York, United States of America; 3 Department of Pathology, University of California, San Francisco, San Francisco, California, United States of America; Duke University, United States of America

## Abstract

Correlative analysis of molecular markers with phenotypic signatures is the simplest model for hypothesis generation. In this paper, a panel of 24 breast cell lines was grown in 3D culture, their morphology was imaged through phase contrast microscopy, and computational methods were developed to segment and represent each colony at multiple dimensions. Subsequently, subpopulations from these morphological responses were identified through consensus clustering to reveal three clusters of round, grape-like, and stellate phenotypes. In some cases, cell lines with particular pathobiological phenotypes clustered together (e.g., ERBB2 amplified cell lines sharing the same morphometric properties as the grape-like phenotype). Next, associations with molecular features were realized through (i) differential analysis within each morphological cluster, and (ii) regression analysis across the entire panel of cell lines. In both cases, the dominant genes that are predictive of the morphological signatures were identified. Specifically, PPARγ has been associated with the invasive stellate morphological phenotype, which corresponds to triple-negative pathobiology. PPARγ has been validated through two supporting biological assays.

## Author Summary

Cell culture models are an important vehicle for understanding biological processes and evaluation of therapeutic reagents. More importantly, the literature suggests that tumor cells grown in 3D exhibit pronounced drug and radiation resistances that are remarkably similar to that of tumors *in vivo*. Therefore, the needs for quantifying 3D assays continue to grow. In this paper, we develop robust computational methods to integrate morphometric and molecular information for a panel of breast cancer cell lines that are grown in 3D. Specifically, morphometric traits are imaged through microscopy, and then quantified computationally. We then show that these morphometric traits can identify subtypes within this panel of breast cancer cell lines, and that the subtypes are clinically relevant in terms of being ERBB2 positive or triple negative. These subtypes and their representations are then associated with their molecular data to reveal PPARG as an important marker for triple-negative breast cancer. Finally, we design two independent experiments to show the validity of this marker in both 3D cell culture models and human breast cancer tissue.

## Introduction

Genome-wide association studies of expression and clinical data have emerged as a powerful methodology for identifying biomarkers of human diseases. While the literature is rich with supervised or unsupervised clustering of genomic information [1], methods for studying the relationships between genomic and physiological responses remain limited. This paper contributes to computational protocols for associating morphometric data, collected through phase contrast microscopy, with genome-wide gene expression data. While genome-wide array expression data provide on average a few readouts with structured measurements for an ensemble of colonies, imaging provides one readout per colony and captures the inherent heterogeneity of a population. However, images are composed of unstructured data that require detailed segmentation and representation for the underlying samples. The net result is subtyping, based on computed morphometric features, and a list of associated genes against computed morphometric features for further bioinformatics analysis. This paper also demonstrates that some of the predicted genes are biologically relevant and can be tested through both *in vitro* and *in vivo* models.

Most of the existing methods for clustering (e.g., subtyping) concentrate on either finding subpopulations for a collection of “OMIC” data or identifying groups of genes that can be associated for each subtype. These methods relate a specific signal across measured conditions, which is appropriate for a focused experiment with a small number of conditions, and for partitioning genes into disjoint sets, thus oversimplifying biological systems. More effective clustering methods have focused on bi-clustering [2]–[4], where bi-clustering aims to find a subset of genes that behave similarly across a subset of conditions. Still, a more effective method is to correlate expression data with known pathways, because pathways represent higher-level biological functions, where the correlation of real value data with known non-numeric pathway data (e.g., KEGG, BRITE) is generally performed through kernel canonical correlation analysis (KCCA) [5]. The original canonical correlation analysis (CCA), developed by Hotelling in 1936, finds projections from two real-value datasets so that those projections have maximum correlations. The kernelized version extends the CCA to non-numerical values. With respect to the understanding of the mechanism of genome-wide regulation and functions, experiments have to be coordinated with the computational requirements to ensure the robustness of any biological conclusion. This is often met by varying or perturbing experimental conditions (e.g., multiple cell lines, different treatment conditions). For example, in a recent paper, microarray data were analyzed with the corresponding physiological responses and clinical metadata [6]. The experiment incorporated NCI-60, a panel of cancer cell lines, that were incubated with Docetaxel, and the impact of the drug was characterized with GI50 (e.g., 50% growth inhibition dose concentration in a 48-hour assay). Subsequently, genes strongly correlated with GI50 were identified.

Experimentally, our method is based on three-dimensional cell culture models, which introduce new computational opportunities, because the assays were imaged with phase contrast microscopy. One primary rationale for designing experiments in 3D cell culture models is that the 3D systems provide a more faithful replication of cell behavior *in vivo* than 2D substrata systems [7],[8]. Mammary cells cultured on rigid 2D substrata rapidly lose many aspects of their *in vivo* phenotype [9], but the use of 3D extracellular matrix cultures (which restore the physiological cell-ECM interactions) allow for a much more faithful replication of *in vivo* phenomena in culture. For example, mammary epithelial cells form polarized acini that vectorially secrete the milk protein, beta-casein, when cultured within a 3D ECM gel [7], and breast cancer cells can be readily distinguished phenotypically from non-malignant breast cells simply by observing their aggressive growth in these assays [10]. Our experiment consists of 24 cell lines from a panel of non-malignant and malignant breast cell lines. We have developed a computational protocol that quantifies colony structures through segmentation and multidimensional representations. Such a multidimensional representation enables subsequent associations with expression data, as well as with the identification of subpopulations among all the 24 lines.

**Figure 1:**
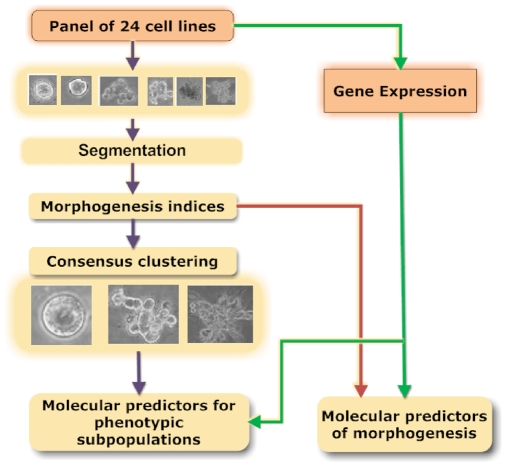
Computational pipeline for differential and association studies between colony morphologies and gene profiles for the panel of breast cancer cell lines cultured in 3D.

Our proposed computational protocol consists of five major steps: (i) colony segmentation, (ii) morphological feature extraction, (iii) consensus clustering of morphological features, (iv) differential analysis of morphological clusters with gene expression profiles, and (v) association of cell-line-specific morphological features and their gene expression signatures. These computational steps are shown in Figure 1, where colonies in each phase image are segmented from the background based on texture features. Regions containing individual colonies are extracted and subsequently represented by multidimensional indices, such as size and Zernike moments. Such a representation is translation and rotation invariant. At this point, one path allows genes that are predictive of morphogenesis to be identified. The second path identifies subpopulations through a modified consensus clustering, which finally leads to ranking those genes that differentiate each subpopulation. A few of these genes are druggable targets, and one has been selected for biological validation.

## Results

### Identification of sub-populations for the panel of cell lines

Our data set includes 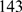 phase images from 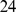 breast cancer cell lines grown in 3D. This data set has produced 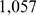 colonies from all 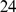 cell lines. Following segmentation and feature extraction, each colony is represented with a multidimensional vector as discussed in the Methods section. This is followed by consensus clustering where the number of clusters is varied from 2 to 7 to examine near optimum partitioning.

**Figure 2:**
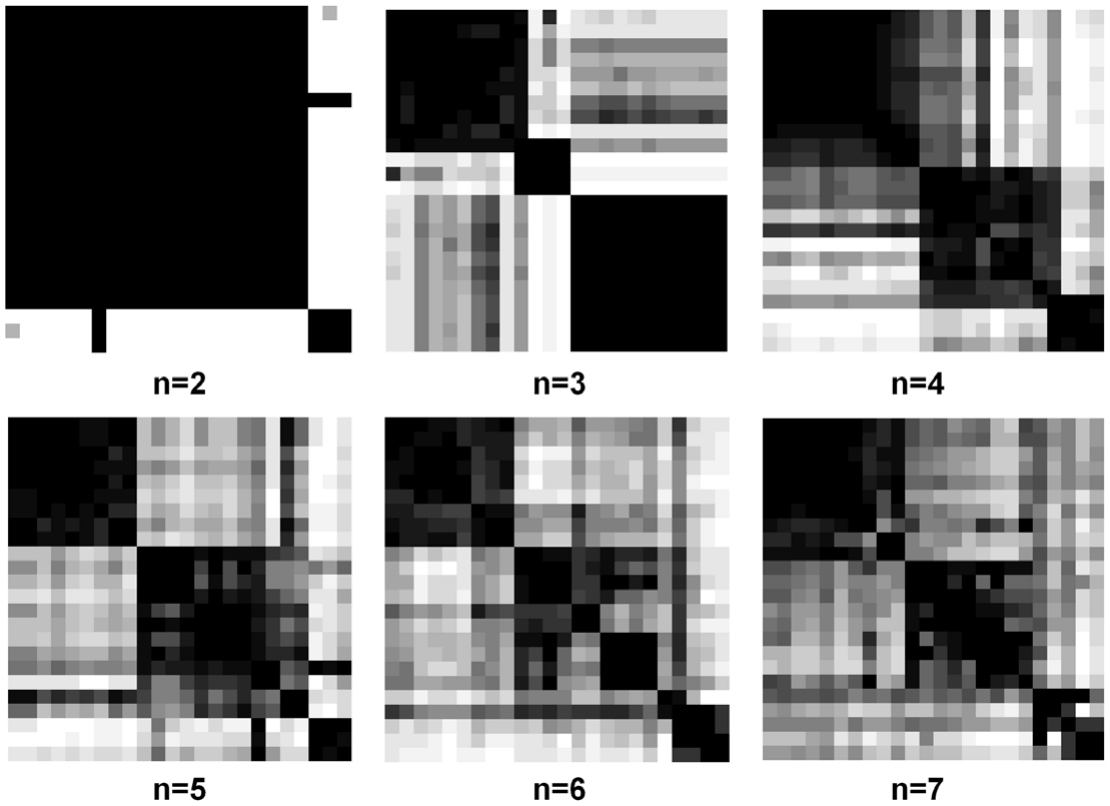
The consensus matrices for different numbers of clusters 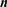 based on morphological representations are shown. A darker block indicates higher morphological similarity between two cell lines. One can hypothesize that the larger block for 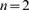 has been partitioned into two blocks for 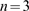; however, the order is not preserved.

**Figure 3:**
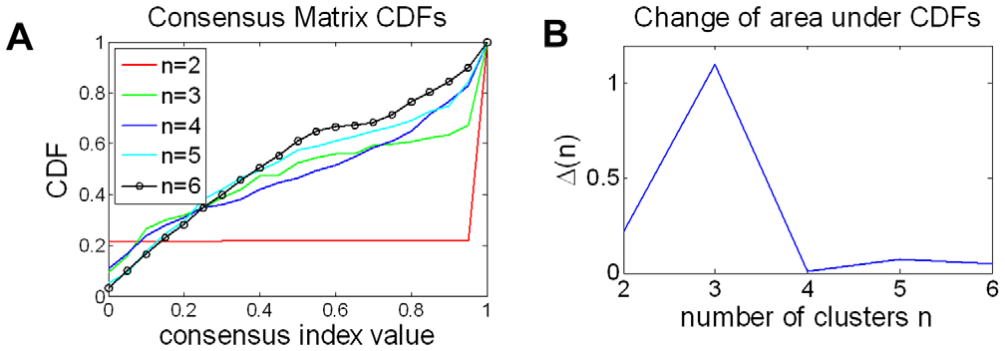
The CDF and its derivative, computed from the consensus matrix, is used to identify the number of clusters. (A) CDF for each cluster, and (B) change in CDF as a function of cluster size, indicates that three is the optimum number of sub-populations.

**Figure 4:**
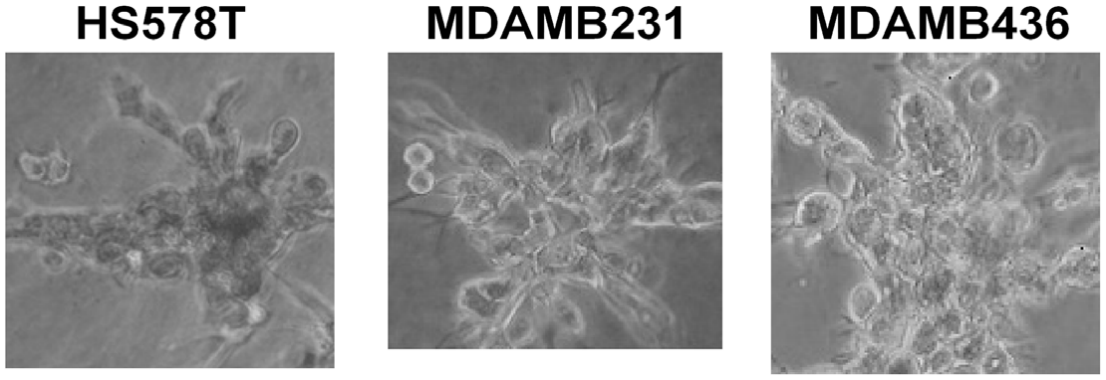
Three cell lines displaying aggressive phenotypes are discovered with 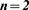. All other cell lines are grouped in a different subpopulation.

**Figure 5:**
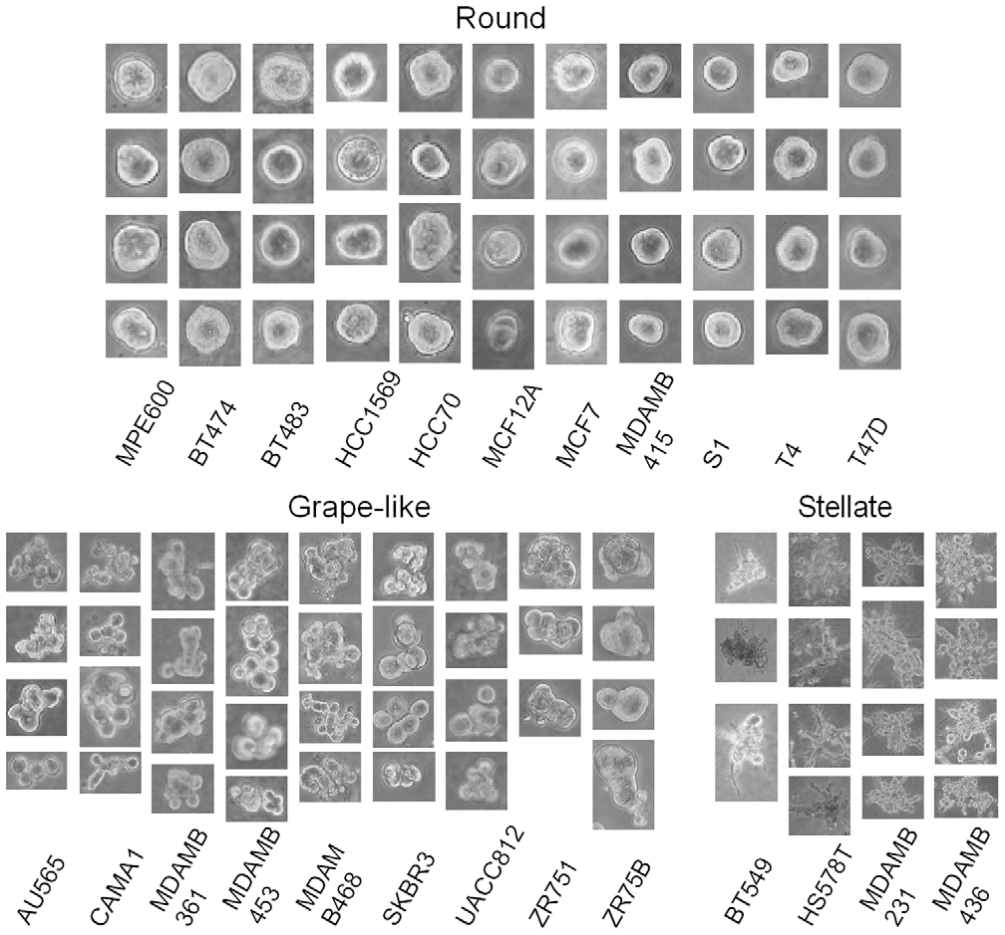
Three subpopulations of the 24 breast cancer cell lines grown in three-dimensional cell culture assay are revealed through consensus clustering.

In order to visualize clustering results, the consensus matrix is traditionally treated as a similarity matrix and reordered using hierarchical clustering. As a result, self similar signatures are placed in close proximity. In this reordered consensus matrix, cell lines with similar morphologies are adjacent to each other, and the darker signal (in the map) reflects improved similarity for the purpose of visualization. Ideally, for a perfect consensus matrix, the displayed heat map should have crisp boundaries. These matrices are generated for a number of clusters, ranging from 2 to 7; the results are shown in Figure 2. The choice of maximum cluster number (e.g., 7) is arbitrary, and the experiment can be repeated if computed consensus matrices and subsequent analysis suggested a larger number of subtypes, but this is biologically less feasible as one is interested in the simplest partition. Consensus clustering assesses stability for the identification of potential subpopulations, and provides visual feedback as a potential component for the decision-making process. For example, for 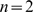, the consensus matrix has one large and one small block with crisp boundaries; and for 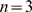, it appears that the large block for 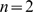 has been partitioned into two other blocks. Therefore, a quantitative method for assigning confidence to the selected number of clusters is needed. This is based on computing consensus distribution [11]. By computing a cumulative distribution from consensus matrices and evaluating proportional increase as a function of the number of clusters, the shape of the concentration distribution can be examined. The cumulative distribution function (CDF) is computed from the entire consensus matrix, whose elements are between 0 and 1. The shape of the CDF and its progression as a function of increase in the number of clusters suggest the presence of desirable subpopulations. An earlier paper by [11] evaluated this method with synthetic and real data, proposed a new measure, a “concentration histogram” computed from the change in the shape of the CDF, and suggested that the peak in the concentration histogram corresponds to an estimate of the number of clusters. The concentration histogram of Figure 3 suggests that three clusters best represent the desired number of subpopulations. Let's examine identification of subpopulations as the number of clusters increases. At 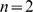, one subpopulation contains three cell lines of 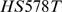, 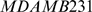, and 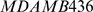, as shown in Figure 4, where their fingerprints indicate large colony size and complex texture representation displaying aggressive behaviors. At 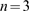, the larger block of 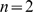 is approximately partitioned into two subpopulations. One subpopulation corresponds to a round symmetrical morphology expected from non-transformed 3D cell culture models. The other population corresponds to a more aggressive line labeled “grape-like” in the literature [12]. In summary, the three clusters of round, grape-like, and stellate, shown in Figure 5, suggest the best set of subpopulations, based on morphological similarities. At 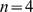 spurious clusters (not shown here) are generated that have no clear boundaries.

### Molecular predictors of phenotypes

Examining the association between phenotypic signatures and expression data is an exploratory step, which requires molecular diversity in the data set to avoid homogeneity. Two distinct approaches are applied, where each approach brings a unique view to the data. (I) In the first approach, expression data associated with each cell line are grouped into their corresponding morphological cluster. As a result, genes that best discriminate between different clusters can be ranked according to their differential strength. (II) In the second approach, genes are ranked against each morphological feature through linear or nonlinear regression analysis. As a result, molecular predictors for positive or negative correlation can be inferred.

#### Molecular predictors of morphological subpopulations

In this case, expression data associated with each cell line are assigned to their own morphological cluster. The objective is to identify genes that best discriminate each morphological cluster. Accordingly, genes are ranked for three classification experiments, where each experiment is one class versus the other two. The main objective is to identify those genes that best predict round, grape-like, and stellate clusters. Table 1 lists those genes, with false discovery rate (FDR) of less than 0.001, that best discriminate stellate versus the other two classes. Similar experiments for round versus stellate and grape-like or grape-like versus stellate and round did not reveal any gene with an FDR of less than 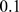. Hence, results are not reported here.

**Table 1:** Best genes for predicting the stellate cluster based on moderated t-statistic (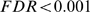).

Gene symbol	Gene description	p-value	FDR	Expression level
PPARG	peroxisome proliferator-activated receptor 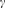	9.13E-15	9.54E-11	+
PARG	poly (ADP-ribose) glycohydrolase	4.31E-09	1.73E-05	+
FADS1///FADS3	fatty acid desaturase 1 /// fatty acid desaturase 3	7.20E-09	1.73E-05	+
PALM2-AKAP2	paralemmin 2 - A kinase (PRKA) anchor protein 2	8.23E-09	1.73E-05	+
FADS3	fatty acid desaturase 3	8.66E-09	1.73E-05	+
TFPI	tissue factor pathway inhibitor (lipoprotein-associated coagulation inhibitor)	1.08E-08	1.73E-05	+
AKAP2/// PALM2/// PALM2-AKAP2	A kinase (PRKA) anchor protein 2 /// paralemmin 2 /// PALM2-AKAP2	1.16E-08	1.73E-05	+
LEPRE1	leucine proline-enriched proteoglycan (leprecan) 1	1.48E-07	1.80E-04	+
DAB2	disabled homolog 2, mitogen-responsive phosphoprotein (Drosophila)	1.74E-07	1.80E-04	+
VCL	vinculin	1.76E-07	1.80E-04	+
PALM	paralemmin	1.90E-07	1.80E-04	+
PTGER4	prostaglandin E receptor 4 (subtype EP4)	2.87E-07	2.50E-04	+
CLCN6	chloride channel 6	3.84E-07	3.09E-04	+
DCBLD2	discoidin, CUB and LCCL domain containing 2	5.36E-07	4.00E-04	+
FSTL1	follistatin-like 1	9.66E-07	6.73E-04	+
FST	follistatin	1.39E-06	9.10E-04	+

#### Molecular predictors of morphological features

Both linear and nonlinear prediction models are explored for molecular predictors of morphological features. Although the simplicity of a linear relationship is quite desirable, many naturally occurring biological interactions are nonlinear. The analysis pipeline has three components: (i) predicting genes that positively correlate with a specific morphological features, (ii) predicting genes that negatively correlate with the same morphological features, and (iii) validating data with a functional analysis. In steps (i) and (ii), a correlation coefficient is transformed into a p-value through permutation analysis.

Tables 2 and 3 summarize the top genes that best predict the size of the colony for positive and negative correlation, respectively, with 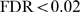.

**Table 2:** Genes that best predict the size of the colony in terms of positive logistic relationship (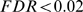).

Gene symbol	Gene description	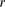	p-value	FDR
PPARG	peroxisome proliferator-activated receptor 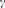	0.8667	1.82E-07	2.18E-03
LPIN2	lipin 2	0.8450	7.49E-07	4.48E-03
VCL	vinculin	0.8145	3.95E-06	1.18E-02
INSIG1	insulin induced gene 1	0.7932	1.06E-05	1.41E-02
APBA2	amyloid beta (A4) precursor protein-binding, family A, member 2 (X11-like)	0.7884	1.31E-05	1.42E-02
CDH11	cadherin 11, type 2, OB-cadherin (osteoblast)	0.7860	1.45E-05	1.45E-02
DLC1	deleted in liver cancer 1	0.7890	1.28E-05	1.53E-02
PRR3	proline rich 3	0.7940	1.03E-05	1.54E-02
BCAT1	branched chain aminotransferase 1, cytosolic	0.7788	1.96E-05	1.67E-02
AXL	AXL receptor tyrosine kinase	0.7697	2.81E-05	1.87E-02
RFTN1	raftlin, lipid raft linker 1	0.796	9.41E-06	1.88E-02
TMEM22	transmembrane protein 22	0.7705	2.73E-05	1.92E-02
AP1S2	adaptor-related protein complex 1, sigma 2 subunit	0.7718	2.59E-05	1.93E-02

**Table 3:** Genes that best predict the size of the colony in terms of negative logistic relationship (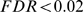).

Gene symbol	Gene description	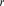	p-value	FDR
F11R	F11 receptor	−0.7162	1.78E-04	2.18E-03
ARHGEF5	Rho guanine nucleotide exchange factor (GEF) 5	−0.7078	2.29E-04	4.48E-03
ITPKC	inositol 1,4,5-trisphosphate 3-kinase C	−0.7019	2.72E-04	9.50E-03
DSP	desmoplakin	−0.6997	2.90E-04	1.18E-02
CTAGE4	CTAGE family, member 4	−0.6692	6.60E-04	1.41E-02
MKRN1	makorin ring finger protein 1	−0.6639	7.55E-04	1.42E-02
HOXC13	homeobox C13	−0.6662	7.12E-04	1.53E-02
SRCAP	Snf2-related CREBBP activator protein	−0.6709	6.32E-04	1.54E-02
PTPLB	protein tyrosine phosphatase-like (proline instead of catalytic arginine), member b	−0.6567	9.02E-04	1.63E-02
LIMK2	LIM domain kinase 2	−0.647	1.14E-03	1.67E-02
RARG	retinoic acid receptor, gamma	−0.6712	6.26E-04	1.72E-02
TNK1	tyrosine kinase, non-receptor, 1	−0.6415	1.29E-03	1.87E-02
MAPRE3	microtubule-associated protein, RP/EB family, member 3	−0.6719	6.15E-04	1.88E-02
OVOL2	ovo-like 2 (Drosophila)	−0.6419	1.28E-03	1.92E-02
CD79A	CD79a molecule, immunoglobulin-associated alpha	−0.6422	1.27E-03	1.93E-02
EPHB3	EPH receptor B3	−0.6462	1.16E-03	1.96E-02

## Discussion

### Morphological subtyping

We compare clustering results with those from interactive methods and provide an interpretation of the morphological similarities based on their known molecular predictors. In an earlier paper [12], an extended set of similar data was analyzed manually, and four subpopulations – round, mass, grape-like, and stellate– were labeled. However, manual analysis of individual colonies is extremely laborious and prone to user bias. Thus, we have developed a computational protocol to identify subpopulations. In our analysis, round and mass clusters are grouped together, since they have no morphological differences when imaged through phase contrast microscopy. However, the above two phenotypes can be differentiated from each other under fluorescence microscopy. The difference is due to the degree of internal organization in these phenotypes. Round colonies tend to have cells arranged in an approximately radial symmetry, while mass colonies are significantly more disorganized. This can only be visualized at higher magnification and confocal microscopy; however, these data have not been included in our analysis. Otherwise, Figure 5 is consistent with Table 1 in [12].

Results indicate that 8 out of 9 cell lines from the grape-like subpopulation express high levels of ERBB2 as a result of amplification of this gene [12], which is differentially expressed between grape-like and round/stellate cell lines with p-value of 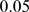. The exception is MDA-MB-468, which has a significant amplification of EGFR. Collectively, these data suggest that the deregulation of signaling through the EGFR/ERBB2 signaling axis may make a strong contribution to the grape-like morphology in culture. The stellate colonies are all negative for estrogen receptors, progesterone receptors, and HER2, a phenotype termed triple negative by pathologists and characterized by a very poor prognosis in cancer patients, as this type of tumor is highly invasive [13]. The invasive nature of the colonies formed by these cells in the 3D culture assay may be reflective of the *in vivo* invasive capacity of these tumor cells.

### Molecular predictors of phenotypic response

Previous results for molecular predictors of morphological subpopulations indicate that the gene expression profiles of stellate colonies are the most distinct from the other two morphological classes, which is consistent with their invasive mesenchymal phenotype compared to the more epithelial colonies formed by round and grape-like cells. A brief description of the molecular predictors, listed in the previous tables, and their relevance is provided below.

Consistent with the mesenchymal phenotype of these cells, PPAR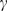, the top gene on this list (Table 1), has been reported to be a potent inducer of EMT in intestinal epithelial cells [14]. Similarly, DAB2 has been reported to be required for TGF-beta induced EMT [15]. PPAR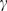 is a nuclear receptor protein, and functions as a transcription factor. It is (i) regulated by thiazolidinediones (TZD), a class of oral anti-diabetic drugs, (ii) involved in proliferation and differentiation [14], and (iii) shown to be highly expressed in metastasized human breast tissue [16]. FADS1 is involved in the synthesis of highly unsaturated fatty acids such as arachidonic acid [17], which (i) are metabolites that activate PPAR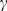 [18], and (ii) can also be converted to prostaglandins, by cyclooxygenases. The Prostaglandin EP4 receptor (PTGER4) was correlated highly with the stellate phenotype and has been implicated in migration of MDA-MB-231 cells *in vitro* [19]. Inhibition of EP4 has been demonstrated to have anti-metastatic effects in preclinical mouse models [20]. Poly(ADP-Ribose) glycohydrolase (PARG) was also highly expressed in stellate cells. PARG and PARP have been reported to localize to sites of DNA damage (reviewed in [21]) and, intriguingly, mice deficient in PARG are hypersensitive to both 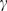-irradiation and alkylating agents [22], suggesting that high levels of PARG may contribute to resistance to DNA-damaging agents in cancer therapy. Stellate cell lines also expressed relatively high levels of Tissue Factor Pathway Inhibitor (TFPI), which is found at high levels in patients with advanced cancer, yet has been proposed to have anti-angiogenic and anti-metastatic functions [23]. Multiple probes corresponding to PALM2/AKAP2, which are alternative splicing variants of the same gene [24], were upregulated in stellate cells. Although the function of PALM2 is not known, PALM1 has been implicated in the filopodia and spine formation during dendritic branching [25], so it is tempting to speculate that PALM2 may contribute to the production of the stellate processes seen in these cell lines. DCBLD2 is highly expressed by metastatic cells in culture, and in lung cancer tissue at both primary and metastatic sites [26].

PPAR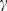 was also the gene most strongly associated with colony size (Table 2). Also highly associated was INSIG1, a PPAR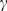 target gene [27], suggesting that the upregulated PPAR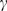 is functionally active in these cells. Axl kinase levels also positively correlated with colony size. Consistent with this, Axl activity has been shown to augment MDA-MB-231 xenograft growth in mammary fat pads and subsequent lung metastasis [28]. Of the other genes associated with colony size, TMEM22 has been reported to play a role in cell proliferation in renal cell carcinoma [29].

Among the genes negatively associated with colony size (Table 3), there are several tumor suppressor genes with roles in normal mammary epithelium. F11R encodes the Junction Adhesion Molecule A (JAM-A) gene. This gene is highly expressed in normal mammary epithelium, but down-regulated in invasive breast cancer cells [16]. TNK1, OVOL2, and EPHB3 are candidate tumor suppressor genes. Deletion of TNK1 in mice results in spontaneous tumorigenesis in several tissues [30]. OVOL2 is a suppressor of c-MYC, and OVOL2-depletion by siRNA promotes cell proliferation [31]. Overexpression of EPHB3 in colorectal cancer cells inhibited proliferation in monolayer culture and growth in both soft agar assays and as xenografts [32].

### Validation

Our validation strategy has two supporting components of *in vitro* and *in vivo* experiments focusing on PPAR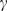, since it is a druggable target. PPAR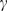 is a hub for lipid metabolism and has been suggested as a therapeutic strategy for epithelial tumor types [33].

**Figure 6:**
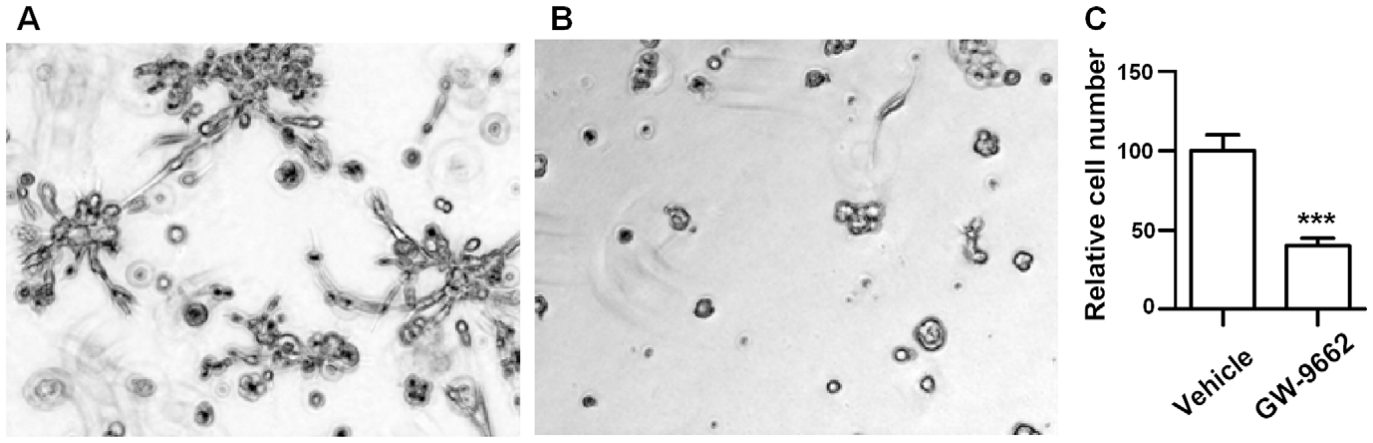
Treatment of a MDA-MB-231 with a PPARG-inhibitor indicates reduction in the proliferation rate. (A) untreated line, (B) treatment with Gw-9662, and (C) proliferation index. The proliferation index was determined by incubating cultures with cell proliferation analysis reagent, WST1, on Day 5.

Figure 6 shows an example of vehicle control, treatment with PPAR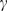 inhibitor, and reduction in the proliferation rate, as measured by the rate of metabolism of WST1. This result is consistent with earlier reports in 2D culture [34] that GW9662 inhibited cell growth and the survival of MDA-MB-231.

**Figure 7:**
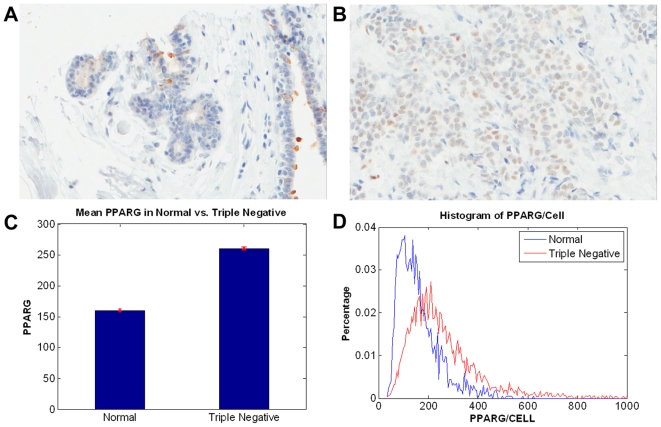
PPARG is expressed in triple negative human breast cancer tissue. (A–B) Localization of PPAR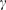 in normal and triple negative of human mammary tissue sections indicates that (i) in normal tissue, localization is apical and unbound to the nuclear regions, and (ii) in triple negative tissue, localization is nuclear-bound and heterogeneous. (C–D) Quantitative analysis on a cell-by-cell basis indicates that PPAR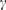 (i) is upregulated in triple negative patients, and (ii) has a heterogeneous distribution.

In the second case, localization of PPAR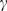 was analyzed by immunohistochemistry in normal breast tissue and in sections from triple-negative breast tumors. Other researchers [35] have examined PPAR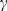 expression in a large cohort of breast tumors, although they did not specifically analyze triple-negative tumors in their studies. Results are shown in Figure 7, and details are included in Text S1.

## Methods

### Cell lines and their culture conditions in 3D

A panel of 24 breast cancer cell lines was cultured in 3D [12]. HMT-3522 S1 (S1) and HMT-3522 T4-2 (T4) mammary epithelial cells were maintained on tissue culture plastic [36]–[39]. The following human breast cancer cell lines were maintained on tissue culture plastic in the following manners: CAMA-1, Hs578T, MCF-7, MDA-MB- 231, MDA-MB-361, MDA-MB-415, MDA-MB-436, MDA-MB-453, MDA-MB-468, MPE-600, SK-BR-3, and UACC-812 were propagated in DMEM/H-21 (Invitrogen) with 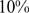 fetal bovine serum (Gemini); AU565, BT-474, BT-483, BT-549, HCC70, HCC1569, T-47D, ZR-75-1, and ZR-75-B were propagated in RPMI 1640 (Invitrogen) with 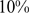 fetal bovine serum; and MCF-12A was propagated in DMEM/F-12 (Invitrogen) with 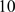 ng/ml insulin, 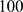 ng/ml cholera toxin, 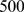 ng/ml hydrocortisone, 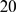 ng/ml EGF (Sigma), and 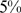 fetal bovine serum. Three-dimensional laminin-rich extracellular matrix (3D lrECM) on-top cultures [40] were prepared by trypsinization of cells from tissue culture plastic, seeding of single cells on top of a thin gel of Engelbreth-Holm-Swarm (EHS) tumor extract (Matrigel: BD Biosciences; Cultrex BME: Trevigen), and the addition of a medium containing 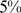 EHS. Cell lines with round 3D morphology were seeded at a density of 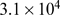 cells per 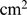; cell lines with stellate 3D morphology were seeded at 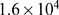 cells per 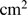; and all other cell lines were seeded at 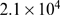 cells per 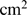. All 3D lrECM cell cultures were maintained in H14 medium with 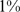 fetal bovine serum, with the exception of S1 and T4, which were maintained in their propagation medium, for 4 days with media change every 2 days.

### Image acquisition and RNA collection

(I) Cell lines were grown in 3D, and cultured colonies were imaged with phase contrast microscopy at 10×. Colonies were isolated from 3D cultures by dissolution in PBS/EDTA [40]. (II) Purified total cellular RNA was extracted using an RNeasy Mini Kit with on-column DNase digestion (Qiagen). RNA was quantified by measuring optical density at A260, and quality was verified by agarose gel electrophoresis. Affymetrix microarray analysis was performed using either the Affymetrix high-density oligonucleotide array human HG-U133A chip cartridge system or the Affymetrix High Throughput Array (HTA) GeneChip system, in which HG-U133A chips were mounted on pegs arranged in a 96-well format. Robust multi-array analysis (RMA) was performed to normalize data collected from different samples. The details can be found in an earlier paper [12]. For gene expression data, the sample size is small, and on the average, there are two samples per cell line. Replicates are either averaged or their medians are selected for representation. On the other hand, the sample size for image-based data is quite large, on the order of thousands.

### Multidimensional profiling of colony morphologies

The first step in multivariate profiling is the segmentation of a colony from its immediate background. Segmentation enables the feature-based representation of each colony for subsequent clustering and correlation analysis with expression data.

#### Colony segmentation

A robust method for delineating samples imaged through phase contrast microscopy is through a bank of gradient feature detectors at different scales and orientations. The main advantage of a multiscale approach is that proper scale (e.g., neighborhood support for computation of the derivative and its orientation) is not known in advance. One immediate consequence of this procedure is that ambiguities due to single-point operations (e.g., thresholding) can be overcome in favor of a more robust process. One implementation of multiscale derivative computation is through a bank of Gabor filters. From the perspective of a mammalian visual system, Gabor filters have the same characteristic as cells in the visual cortex. From a computational perspective, these filters have been shown to have an optimal localization in both spatial and frequency domains [41], and the filter bank can be designed so that the overlap between individual filters is minimized.

**Figure 8:**
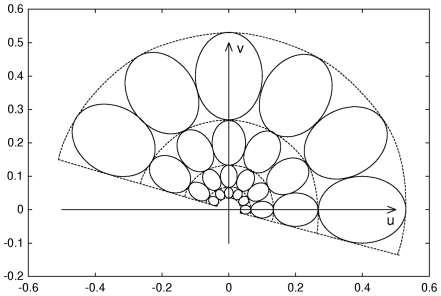
The elliptical contours indicate the half-peak magnitude iso-curves of the Gabor filters, in the frequency domain, at 6 orientations and 4 scales. At each scale, mean filter response is invariant to rotation.

In our implementation, rotation-invariant Gabor features are used to characterize image gradient information at different scales [42]. A 2D Gabor function 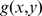 and its Fourier transform 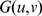 can be expressed as:
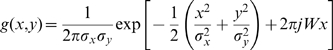
(1)and
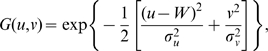
(2)respectively, where 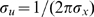 and 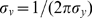. A set of Gabor functions can be generated by rotating and scaling 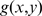. Let 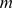 and 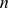 be scaling and rotation parameters, respectively. Then 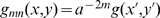, where 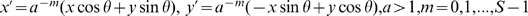, and 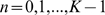. To reduce the redundancy in the filtered images, the filter parameters are chosen to ensure that the adjacent half-peak magnitude iso-curves are tangential to each other in the frequency domain. For example, Fig. 8 shows iso-curves of half-peak magnitude at six different orientations and four scales (e.g., size) [43].

**Figure 9:**
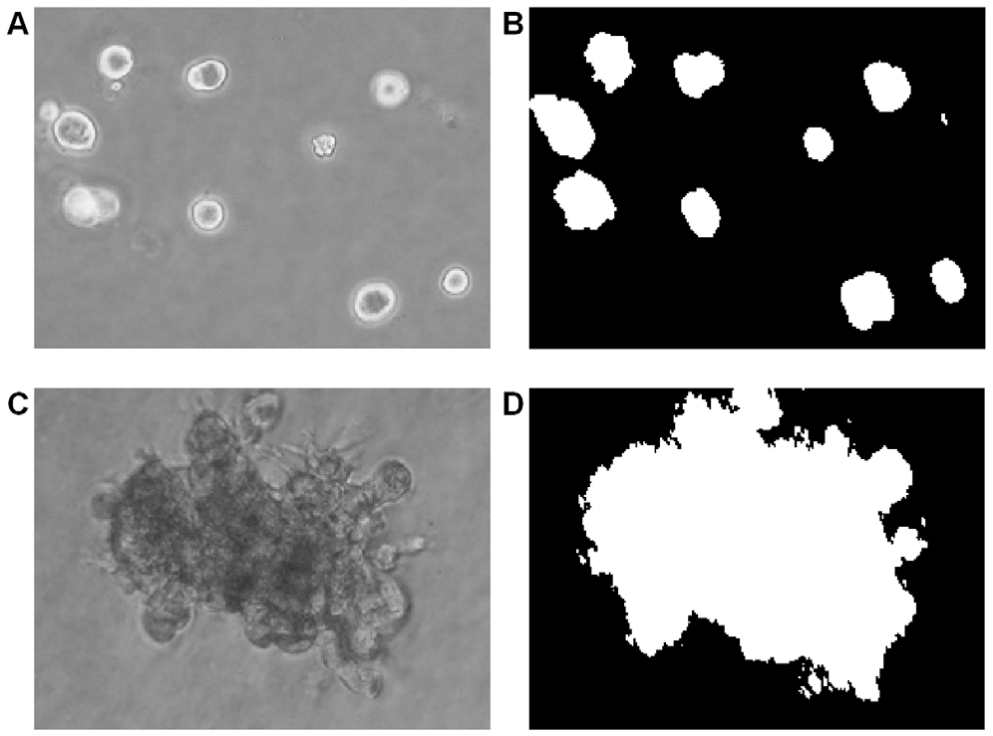
Regions associated with the multicellular colonies are differentiated through proposed computational method. (a)(c) original images of two types of colonies with contrast reversal (e.g., dark regions in the bottom row versus bright regions in the top row), and (b)(d) the corresponding segmented results. Segmentation is feasible as a result of the Gabor filter bank that encodes oriented texture features.

By accumulating all rotated filters (e.g., integration over 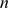), at each scale (e.g., every 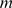), a series of rotation-invariant filters,
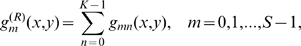
(3)whose entire half-plane frequency spectrum is shown in each dotted region of Figure 8, are obtained. Thus, the filtered images represent rotation-invariant texture properties of the input image, 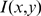,
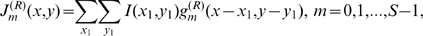
(4)where each pixel is represented by an 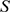-dimensional texture feature vector. Subsequently, the K-means method is used to delineate background and foreground pixels. The initial condition is set at the lowest and highest values of the filter response. The end result is a binary representation of the original image corresponding to one of the two classes; examples are shown in Figure 9.

#### Phenotypic representation

Following segmentation, morphometric properties of each colony are represented for subtyping or clustering. However, such a representation has to be invariant to rotation and translation (e.g., the area of a colony has such a property), since orientation of a colony cannot be predicted a priori [44],[45]. Here, we opted to use Zernike moments, which have been widely used for representation, and are shown to outperform other moment invariants in shape-based classification and recognition [45]. In our system, the first 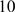 orders of moments are computed from the image gradient, which is invariant to contrast reversal and shading, and then normalized to 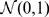. Overall, each segmented colony is represented by a 33-dimensional vector. Zernike moments of 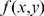 are defined as

[44]

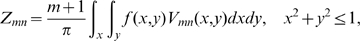
(5)where 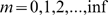, 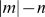 is even, and 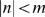, and Zernike polynomials 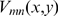 are a set of orthogonal functions. The background material on the Zernike polynomial is included in Text S1.

#### Clustering of phenotypic signatures

Clustering of phenotypic signatures contributes to the categorization of morphological features and to the subsequent correlation analysis of expression data. However, three issues need to be addressed: (i) colonies have heterogeneous morphologies for the same cell line; (ii) the number of colonies for each cell line is unequal, ranging from a dozen to several hundred independent samples; and (iii) there is no prior knowledge of the number of clusters. An important aspect of clustering has to do with validation, since some clustering methods (e.g., k-means) are sensitive to the initial conditions, and others simply quantize the space (e.g., hierarchical clustering) through an arbitrary threshold. A proven method is consensus clustering, which is widely used for class discovery and visualization of gene expression microarray data [46]. This iterative method is based on resampling, and is designed to partition the observed gene expression profiles into a set of exhaustive and nonoverlapping clusters. In each iteration, clustering is performed on a random subset of the data, and the consensus across repeated runs is aggregated into a consensus matrix, which represents the probability that a pair of cell lines will be in the same cluster. Furthermore, visualization of the consensus matrix enables the qualitative evaluation of the clustering results (e.g., Are there crisp boundaries between clusters?). Our goal is to partition morphometric fingerprints of colonies associated with 24 cell lines into a set of exhaustive and nonverlapping clusters. We modified the consensus clustering method slightly:

1. Initialize the number of clusters to 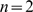.
2. Construct an equal number of samples, 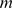 (e.g., colony), from each of the 24 cell lines through random sampling.
3. Cluster 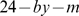 randomly selected samples using the k-means method.
4. Construct a probability distribution function (PDF) for each cell line. This PDF indicates the assignment of samples to each cluster. In other words, each cell line will have its own unique PDF given the number of clusters.
5. Construct the similarity matrix whose elements correspond to the p-value computed through the Kolmogorov-Smirnov (KS), which compares pairwise similarities between two distributions.
6. Repeat steps 2 to 5 for a fixed number of 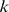 iterations, and compute the consensus matrix (e.g., average or median over all 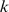 similarity matrices).
7. Increase 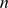 and repeat steps 1 to 6 for each different 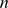.

The KS test is nonparametric, makes no assumption about the distribution of the data, and outputs a 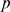 value between two distributions (e.g., 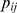). Each element of the similarity matrix 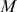 is represented as 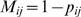, and the final consensus matrix is constructed by averaging all similarity matrices for all 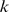 iterations. Subsequent visualization of the consensus matrix enables visual feedback for the performance of the clustering results. In our system, the number of iterations (e.g., 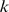) and samples (e.g., 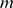) are set at 100 and 6, respectively.

#### Identification of molecular predictors for morphological clusters

We have examined both linear and nonlinear methods for differential expression between different clusters. Additionally, the same biomarker has been identified through gene set enrichment analysis (GSEA) [47]. Results from GSEA and nonlinear analysis are shown in Text S1. Regardless of linear and nonlinear cases, the main challenge is the limited number of gene expression data. However, since the same biomarker has appeared in both cases, we are limiting our discussion to the linear method in the main body of text. In the nonlinear case, the cross-validation error of the SVM rule with Gaussian kernel is used for identifying differentially expressed genes [48]. The details of the nonlinear method are summarized in Text S1.

In the linear case, gene selection is based on the moderated t-statistic [49], which uses the empirical Bayes method for assessing differential gene expression. In the moderated t-statistic, ordinary standard deviations are replaced by posterior residual standard deviations, and the results are further moderated across genes through the empirical Bayes approach. The net result is an improved statistical stability given the limited number of samples. The p-value is computed for each gene based on the moderated t-statistic, and then adjusted for multiple hypothesis testing. The adjustment is based on Benjamini and Hochberg's method to estimate the false discovery rate (FDR) [50]. FDR controls the expected proportion of falsely rejected null hypotheses in multiple hypothesis testing to correct for multiple comparisons. The method is implemented through the R *limma* package [51].

### Identification of molecular predictors for morphological features

Both linear and nonlinear prediction models are explored to identify molecular predictors. Each model produces a different view of the analysis for subsequent biological validation. In linear regression, the relationship between two variables (e.g., morphology index and gene expression) is given by
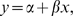
(6)where the coefficients 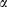 and 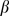 are estimated by minimizing the 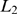 norm (e.g., sum squared error): 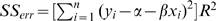, where 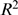 is known as the coefficient of determination in statistics and is the proportion of variability in a dataset that can be accounted for by the model. A general definition is given by the ratio of error in the fit (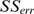) to sample variance (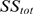):
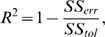
(7)where, as before, 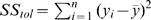. In linear regression, the square root of 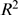 equals the Pearson product-moment correlation coefficient:
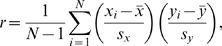
(8)where 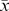 is the sample mean and 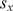 is the sample standard deviation. Therefore, the Pearson product-moment correlation coefficient measures the quality of least squares fitting to 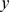 and 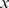 in Equation (6), i.e., the degree of linear relationship between two variables. A value of 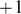 indicates a perfect positive linear relationship, and 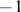 means a perfect negative linear relationship.

In the nonlinear case, the relationship is modeled by a logistic function [52]:
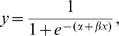
(9)where 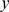 samples are normalized to reside between 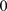 and 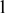. Equation (9) can be rewritten as
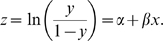
(10)The Pearson product-moment correlation coefficient of the transformed variable 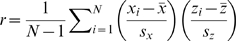
measures the fitting quality of 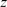 and 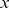 in Equation (10), as well as the quality of the logistic fitting to the original data 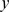 and 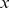 in Equation (9).

In all cases, the p-value is computed through permutation. In each permutation step, a subset of the data is used to compute the corresponding Pearson product-moment correlation coefficients based on a higher-level taxonomy for genes being either positively or negatively correlated with morphogenesis. For each gene, from their respective taxonomy, a p-value is then computed by comparing its Pearson product-moment correlation coefficient 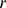 with 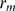 values, 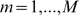, from 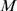 permutated samples. For a gene with positive 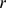 value, its p-value is:
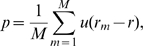
(11)where 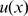 is 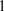 if 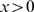, and 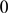 otherwise. For a gene with negative 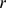 value, its p-value is:
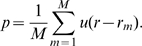
(12)


### Validation

#### *In vitro* approach

In the first case, the triple-negative breast cancer cell line, MDA-MB-231, was assayed in 3D cell cultures maintained in H14 medium with 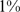 fetal bovine serum. The 3D cultures were prepared in triplicate by seeding single cells on top of a thin layer of Matrigel (BD Biosciences; Franklin Lakes, NJ) at a density of 2,200 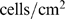 and overlaid by 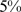 final Matrigel diluted in a culture medium. GW9662 (Cayman Chemical; Ann Arbor, MI), a PPARG inhibitor, was dissolved in DMSO (Fisher Scientific; Hampton, NH) and added to the 3D cultures in the final concentration of 10 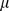
M at the time of seeding. The vehicle control was pure DMSO. The culture medium and the drug were changed every other day. Five images per well were collected after five full days in 3D culture on an Olympus IX 81 (Melville, NY) with 10× N.A. 0.25 Plan APO optics with a Cooke Sensicam QE air-cooled CCD camera, using IPLab 4.0.

#### *In vivo* approach

The PPAR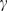 antibody, from the EnVision kit, was initially assayed at 1∶25, 1∶50, 1∶100, and 1∶200 dilution, with 1∶25 (1 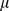
L of antibody per 25 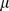
L of buffer) being selected as the optimal dilution. Detection was performed using the Envision System (DakoSytomation). Paraffin-embedded, triple-negative (from three different patients) and normal tissue sections were stained and scanned with an Aperio imaging system at 40×. Since these images have a very large format (e.g., approximately 50,000-by-50,000 pixels), they were randomly sampled for quantitative analysis. Each sampled sub-image is 1472-by-936 pixels, and the amount of nuclear-localized PPAR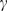 was quantified using a recently published method [53]. All nuclear segmentations were manually corrected to exclude stromal cells based on their morphology.

## Supporting Information

Text S1 This file contains supplementary materials. Section 1 shows how pure thresholding fails in delineating foreground and background. Section 2 provides a summary of Zernike polynomial for representing morphometric traits. Section 3 summarizes background on non-linear regression methods for identifying molecular targets. Section 4 provides comparative analysis with the Gene Set Enrichment Analysis (GSEA). Section 5 outlines the details of validation protocol that includes quantitative image analysis.
(0.78 MB PDF)
