# Supplementary material for: Molecular Predictors of 3D Morphogenesis by Breast Cancer Cell Lines in 3D Culture
Source: PLoS Comput Biol. 2010 Feb 26;6(2):e1000684. doi: 10.1371/journal.pcbi.1000684 (PMC2829039; doi:10.1371/journal.pcbi.1000684)
Supplement: Text S1 — This file contains supplementary materials. Section 1 shows how pure thresholding fails in delineating foreground and background. Section 2 provides a summary of Zernike polynomial for representing morphometric traits. Section 3 summarizes background on non-linear regression methods for identifying molecular targets. Section 4 provides comparative analysis with the Gene Set Enrichment Analysis (GSEA). Section 5 outlines the details of validation protocol that includes quantitative image analysis. (0.78 MB PDF) [file pcbi.1000684.s001.pdf]

Molecular Predictors of 3D Morphogenesis by Breast Cancer Cell Lines in 3D Culture: Supplementary Material

These sections contain supplementary materials. Section 1 shows how pure thresholding fails in delineating foreground and background. Section 2 provides a summary of Zernike polynomial for representing morphometric traits. Section 3 summarizes background on non-linear regression methods for identifying molecular targets. Section 4 provides comparative analysis with the Gene Set Enrichment Analysis (GSEA). Section 5 outlines the details of validation protocol that includes quantitative image analysis.

1 Thresholding as a mean for segmentation

Gabor filters eliminate the need for threshold selection and complexities that may arise because of contrast reversal with phase contrast microscopy. Figure 1 shows three examples of thresholding artifacts in our data sets. However, by utilizing Gabor features, these artifacts can be eliminated.

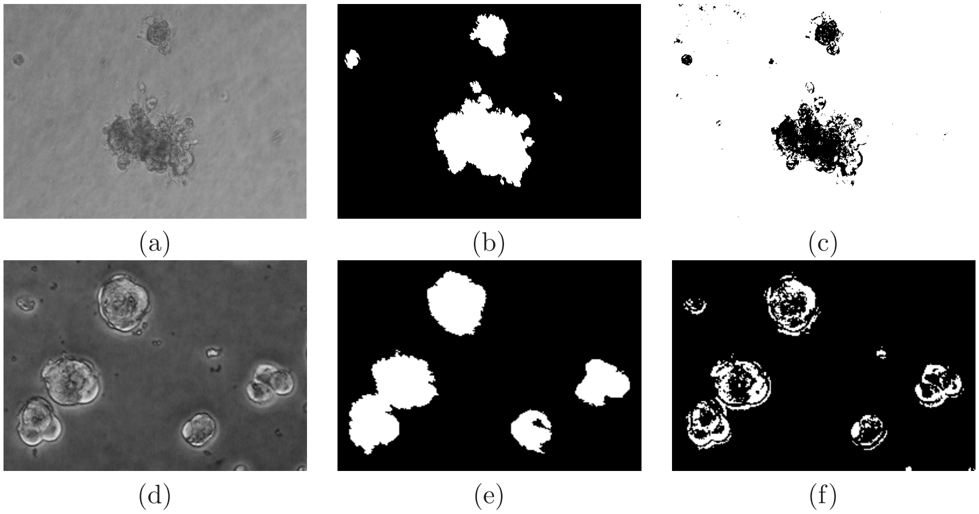

Figure 1: Comparison of thresholding with Gabor filter bank in delineating colonies from background. Clearly, thresholding leaves behind holes and other artifacts.

2 Background on Zernike Polynomial

The Zernike polynomials $V_{mn}(x, y)$ are a set of orthogonal functions that satisfy

$$\int_x \int_y V_{mn}(x,y)^* V_{kl}(x,y)dxdy = \frac{m+1}{\pi}\delta_{mk}\delta_{nl}, \quad x^2+y^2 \leq 1, \tag{1}$$

where δ_{mk} is 1 if $m = k$, and 0 otherwise. Zernike polynomials expressed in polar coordinates (ρ, θ) are defined as

$$V_{mn}(\rho,\theta) = R_{mn}(\rho)e^{jn\theta}, \tag{2}$$

where

$$R_{mn}(\rho) = \sum_{k=0}^{\frac{m-|n|}{2}} (-1)^k \frac{(m-k)!}{k!(\frac{m+|n|}{2}-k)!(\frac{m-|n|}{2}-k)!}\rho^{m-2k}. \tag{3}$$

The significance of such a representation is that they provide a translation and rotation invariant measure to encode inherent morphometric properties.

3 Molecular predictors of morphological clusters based on non-linear method

In the non-linear case, the .632+ bootstrap error [1] of the SVM rule with Gaussian kernel is used for identifying differentially expressed genes. Bootstrap is a resampling method for model selection and validation that is shown to perform well for small sample sizes by correcting the bias against sample selection. As discussed by Ambroise and McLachlan [1], the .632+ bootstrap error is estimated by

$$E_{B.632+} = (1 - w)E_{resub} + wE_{bs}, \tag{4}$$

where E_{resub} is the proportion of original cell lines misclassified by the SVM rule R, constructed from data associated of all cell lines (i.e., the entire data set is used for training); E_{bs} is the leave-one-out bootstrap error rate for predicting the classification error of a specific cell line, which is not included in the bootstrap samples; and w is the weight. Suppose that K bootstrap samples of size n are obtained by re-sampling with replacement from the original N cell lines of known cluster labels. The re-sampling scheme is designed in such a way that each bootstrap sample contains the same number of cell lines from each morphological cluster. E_{bs} in Eq. (4) is then estimated by

$$E_{bs} = \frac{1}{N}\sum_{i=1}^{N} E_i, \tag{5}$$

where

$$E_i = \frac{\sum_{k=1}^{K} O_{ik}E_{ik}}{\sum_{k=1}^{K} O_{ik}}. \tag{6}$$

O_{ik} is 0 if the ith cell line exists in the kth bootstrap sample and is 1 otherwise. $E_{ik} = 1$ if the SVM rule R_k, formed from the kth bootstrap sample, misclassifies the ith cell line, and equals 0 otherwise. The weight w in Eq. (4) is defined by

$$w = \frac{0.632}{1 - 0.368r} \tag{7}$$

where

$$r = \frac{E_{bs} - E_{resub}}{\gamma - E_{resub}} \tag{8}$$

is the relative overfitting rate and γ is the no-information error rate, which is estimated by

$$\gamma = \sum_{i=1}^{c} p_i(1 - q_i), \tag{9}$$

where c is the number of classes or clusters, p_i is the percentage of the cell lines from the ith class with respect to the entire population, and q_i is the correct recognition rate as measured by the SVM rule R.

The top genes selected to predict the stellate cluster based on .632+ bootstrap error of SVM with Gaussian kernel are listed in Tables 1, with annotations.

4 Molecular predictors of morphological clusters based on GSEA

We run GSEA on the gene expression data with the label of stellate vs. round/grape-like. Table 2 shows gene sets (gene ontology terms) enriched in the stellate cluster based on the GSEA results. PPARG appears in 4 of the most enriched gene sets.

Table 1: Best genes for predicting the stellate cluster based on .632+ bootstrap error of SVM with Gaussian kernel ($E_{B.632+} < 1\%$).

Gene symbol	Gene description	$E_{B.632+}$	Expression level
PPARG	peroxisome proliferator-activated receptor gamma	0	+
FADS1///FADS3	fatty acid desaturase 1///fatty acid desaturase 3	0	+
ZEB1	zinc finger E-box binding homeobox 1	0.0013	+
PVRL3	poliovirus receptor-related 3	0.0024	+
AKAP2///PALM2 ///PALM2-AKAP2	A kinase (PRKA) anchor protein 2///paralemmin 2///PALM2-AKAP2	0.0036	+
DOCK10	dedicator of cytokinesis 10	0.0037	+
CLCN6	chloride channel 6	0.0043	+
CTAGE4///LOC100142659 ///LOC441294	similar to CTAGE6///CTAGE family, member 4///CTAGE family member	0.0047	-
DAB2	disabled homolog 2, mitogen-responsive phosphoprotein (Drosophila)	0.0048	+
FLJ10357	hypothetical protein FLJ10357	0.0063	+
PALM2-AKAP2	PALM2-AKAP2	0.0095	+

5 Validation

Kenny's lab has been responsible for validation of PPARγ against the stellate line. Validation against triple negative mammary tissue has been performed by Dr. Baehner, a pathologist. His conclusion is that there is a focal difference in localization of PPARγ between normal and triple negative tissue sections. Nevertheless, we opted to quantify these differences using a recently developed system. In this system, nuclear regions are segmented, and the regions between neighboring nuclei are partitioned through Voronoi tessellation. Next, the brown signal associated with PPARγ is deconvolved from hematoxylin (e.g., nuclear labeling blue signal) through non-negative matrix factorization [2]. Finally, the signals within the nuclear regions are accumulated on a cell-by-cell basis. Intermediate results are shown in Figure 2. Each segmented nuclear reveals a distribution corresponding to PPARγ. These distributions are accumulated for normal and triple negative cells, and results are reported.

References

1. Ambroise C, McLachlan G (2002) Selection bias in gene extraction on the basis of microarray gene-expression data. Proc Natl Acad Sci USA 99: 6562-6566.

2. Rabinovich A, Agarwal S, Laris C, Price J, Belongie S (2003) Unsupervised color decomposition of histologically stained tissue samples. Arch Pathol Lab Med .

Table 2: Gene sets (gene ontology terms) enriched in the stellate cluster based on GSEA results.

GO term	Related genes	NES	p-val	FDR
Positive regulation of cell differentiation	ACIN1,ACVR1B,ACVR2A,ADIG,BMP4,BMPR1B,BOC,BTG1, CALCA,ETS1,FOXO3,IGFBP3,IL20,IL7,INHBA,NME2,**PPARG**, RUNX1,SART1,SCIN,SOCS5,TBX5,TGFB2,VWC2,ZAP70	1.8190	0	0.1928
Contractile fiber	ABRA,ACTA1,CDK5R1,DES,DMD,KRT19,MYBPC1,MYL3, MYL5,MYL6B,MYL9,MYLPF,MYOM1,MYOZ2,NEB,SVIL, TNNC1,TNNI3,TNNT2,TPM1,TPM2,TPM3,TPM4,TTN,VCL	1.7450	0.0045	0.2780
Contractile fiber part	ABRA,ACTA1,DES,DMD,KRT19,MYL3,MYL5,MYL6B,MYL9, MYLPF,MYOM1,MYOZ2,NEB,SVIL,TNNC1,TNNI3,TNNT2, TPM1,TPM2,TPM3,TPM4,TTN,VCL	1.8193	0.0032	0.2881
Response to extracellular stimulus	ALB,ASNS,CARTPT,CCKAR,CDKN1A,CDKN2B,CDKN2D, CHMP1A,ENPP1,ENSA,FADS1,GCGR,GHRL,GHSR,GIPR,GNAI2, LEP,NPY,NUAK2,OGT,PCSK9,**PPARG**,PPP1R9B,RASGRP4,RPS19, SREBF1,SST,SSTR1,SSTR2,STC1,STC2,TP53,TULP4	1.7161	0.0154	0.3069
Basolateral plasma membrane	ACTN1,ACTN2,ACTN3,ATP7A,ATP7B,B4GALT1,BCAR1,BEST1, BSND,C9orf58,CADM1,CLDN19,DLG1,DST,ERBB2IP,EVL,LAYN, LDLRAP1,LIMA1,MET,MUC20,MYO1C,NEXN,NRAP,PTPRC, SLC16A10,SLC4A11,SNIP,SORBS1,SORBS3,STX2,STX4,TJP1, TRIP6,VCL	1.7547	0.0046	0.3160
Response to nutrient levels	ALB,ASNS,CARTPT,CCKAR,CDKN2B,CDKN2D,CHMP1A, ENPP1,ENSA,FADS1,GCGR,GHRL,GHSR,GIPR,GNAI2,LEP, NPY,NUAK2,OGT,PCSK9,**PPARG**,SREBF1,SST,SSTR1,SSTR2, STC1,STC2,TP53,TULP4	1.8270	0	0.5209
DNA dependent atpase activity	BPTF,CHD1,CHD2,CHD3,CHD4,DHX9,ERCC6,ERCC8,G3BP1, PIF1,RAD51,RAD54B,RBBP4,RECQL,RFC3,RUVBL2,SMARCA1, SMARCAL1,TOP2A,TTF2,XRCC5,XRCC6	1.5202	0.0465	0.8447
Positive regulation of response to stimulus	BCAR1,C2,CADM1,CD1D,CD79A,CDH13,CEBPG,CFHR1,CRTAM, CX3CL1,EEF1E1,EREG,FYN,GHRL,GHSR,IFNK,IKBKG,IL12A, IL12B,IL29,IL8,KRT1,LAT2,MALT1,MAP3K7,MBL2,NFAM1, NPY,PRKCG,PTPRC,SCG2,SLA2,SLIT2,TGFB2,THY1,TLR8, TNFRSF1A,TRAF2,TRAF6,TRAT1,UBE2N	1.5007	0.0175	0.8855
Regulation of cell differentiation	ACIN1,ACVR1B,ACVR2A,ADIG,BMP4,BMPR1B,BOC,BTG1, CALCA,CARTPT,CDK6,CNTN4,DTX1,EREG,ETS1,FOXO3,FOXO4, GPR98,IGFBP3,IL20,IL27,IL4,IL7,INHA,INHBA,IQCB1,LDB1, MAFB,MAP4K1,NANOG,NF1,NLGN1,NME2,NOTCH1,NOTCH2, NOTCH4,NPHP3,PF4,**PPARG**,RUNX1,SART1,SCIN,SHH,SNF1LK, SOCS5,SPI1,SPINK5,TAF8,TBX3,TBX5,TCFL5,TGFB2,TWIST2, USH2A,VWC2,YWHAG,YWHAH,ZAP70,ZBTB16,ZNF675	1.5248	0.0101	0.9034
Positive regulation of immune response	BCAR1,C2,CADM1,CD1D,CD79A,CFHR1,CRTAM,EREG,FYN, IFNK,IKBKG,IL12A,IL12B,IL29,KRT1,LAT2,MALT1,MAP3K7, MBL2,NFAM1,PTPRC,SLA2,TGFB2,THY1,TLR8,TRAF2,TRAF6, TRAT1,UBE2N	1.4725	0.0152	0.9184
Extracellular matrix structural constituent	ACAN,CHI3L1,COL4A2,COL4A4,COMP,DSPP,EFEMP2,FBLN1, FBLN2,FBN1,FBN2,IMPG1,IMPG2,KAL1,LAMA1,LAMA4, LAMB1,LAMC1,MATN1,MATN3,MEPE,MFAP5,MGP,MUC2, OPTC,PRELP,TFPI2	1.5304	0.0308	0.9642

Table 3: Expression of PPARγ in 3D vs. 2D in log2 scale. For differential expression between stellate and round/grape-like cell lines in 3D culture, PPARγ ranks as the top gene with p-value of $9.13E-15$ and FDR-adjusted p-value of $9.54E-11$. In 2D culture, PPARγ ranks as the 462-th gene with p-value of 0.0023 and FDR-adjusted p-value of 0.0671.

Subpopulation	Cell line	3D	2D
Round	600MPE	0.3290	-0.1223
	BT474	-0.6718	-0.6213
	BT483	-1.1710	-0.7686
	HCC1569	0.3118	0.0880
	HCC70	-0.6482	-0.3973
	MCF12A	-0.5424	0.2205
	MCF7	-1.1541	0.3275
	MDAMB415	-0.6063	-0.2282
	S1	-0.8628	NA
	T4	-1.2737	NA
	T47D	-0.9862	-0.3399
Grape-like	AU565	NA	-0.2708
	CAMA1	NA	-0.4964
	MDAMB361	-1.2273	-0.2731
	MDAMB453	-0.9527	-0.6809
	MDAMB468	-0.0010	0.3849
	SKBR3	-0.1549	0.0692
	UACC812	1.0200	1.1344
	ZR751	-1.0792	-0.5508
	ZR75B	-0.8879	-0.6201
Stellate	BT549	2.4880	0.3240
	HS578T	2.7509	0.2887
	MDAMB231	2.4872	0.9287
	MDAMB436	2.8415	1.6037

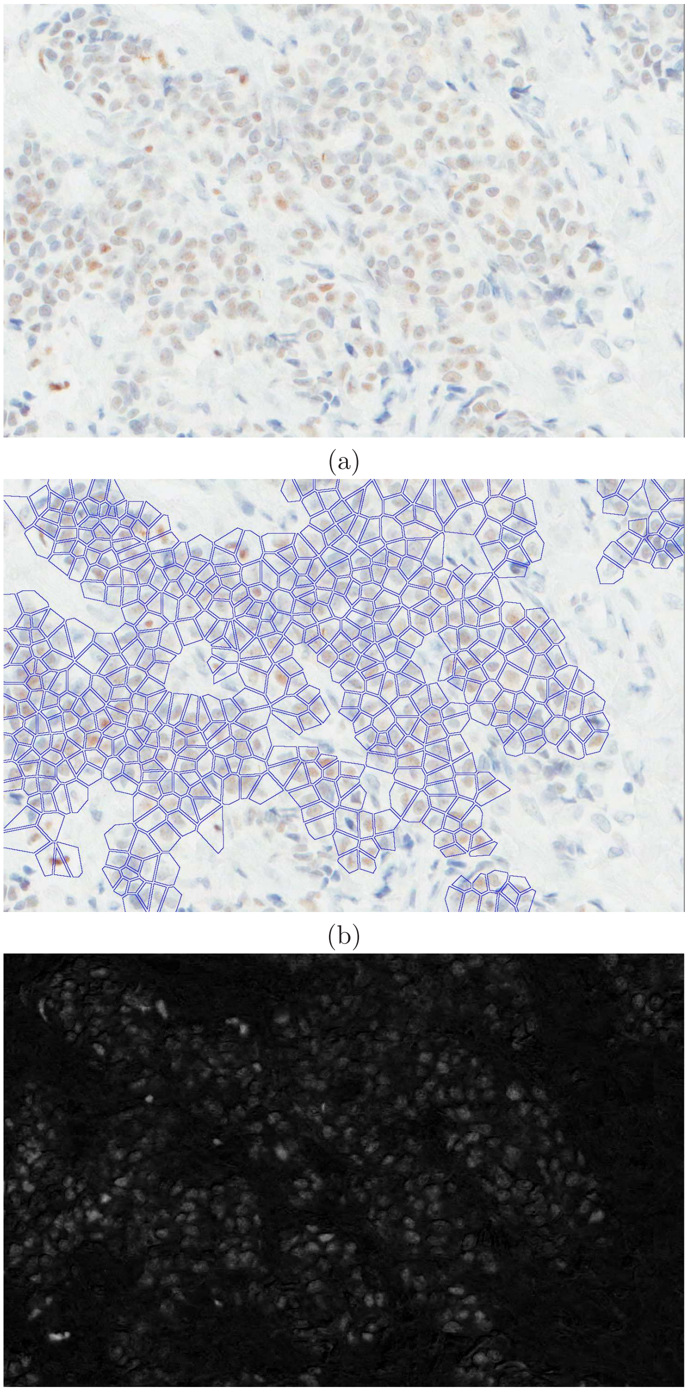

(a)

(b)

(c)

Figure 2: Quantitative analysis of histological sections: (a) original image; (b) Voronoi tessellation following nuclear segmentation, and (c) non-negative matrix factorization corresponding to PPARγ.
